# Supplementary material for: Humans and great apes visually track event roles in similar ways
Source: PLoS Biol. 2024 Nov 26;22(11):e3002857. doi: 10.1371/journal.pbio.3002857 (PMC11593759; doi:10.1371/journal.pbio.3002857)
Supplement: S1 Methods — (DOCX) [file pbio.3002857.s001.docx]

# Supplementary Materials

**Methods**

## Participants

**Nonhuman apes.** Nine great apes from three species were tested. These included five chimpanzees (3 females, age in years: mean = 6.4, SD = 6.14, span = [2.5, 17]), two gorillas (1 female, both 5 years old), and two orangutans (1 female, 15 and 19 years old). The youngest participants included already showed some level of independence from their mothers and interacted freely with their peers. All participants were housed in social groups at Basel Zoo, Switzerland (chimpanzees: 8 adults [2 male], 4 juveniles [2-6 years; 1 male], 1 infant [< 1 year]; gorillas: 5 adults [1 male], 2 juveniles [5 years old; 1 male], 1 infant [< 1 year old]; orangutans: 6 adults [3 male], 3 juveniles [2-7 years; 1 male]). Each group had access to both an indoor and an outdoor enclosure (chimpanzees, indoor: 1096.93 m^3^, outside: 3860.1 m^3^; gorillas, indoor: 1148.84 m^3^, outside: 3025.05 m^3^; orangutans, indoor: 1726.69 m^3^, outside: 8965.1 m^3^).

**Humans.** Fourteen human adults (7 female, age in years: mean = 26.79, SD = 6.73, span = [18, 41]), recruited among the students of the University of Zurich, participated for payment. Twenty-nine human infants (16 female, age in days: mean = 191.79, SD = 11.80, span = [168, 219]) were recruited through the research unit Developmental Psychology: Infancy and Childhood at the University of Zurich. Infants of parents who speak a language with an ergative case system (e.g., Basque or Hindi) were excluded from recruitment because ergative case is known to modulate agent attention (1). Consistent with general drop-out rates for infant studies (2), data from 15 additional infants were excluded from analyses because they had overall poor or missing calibrations, were not attending to the stimuli, or because (in one case) the mother spoke a language with an ergative case marking system.

## Materials

Eighty-four video clips were selected as stimuli to be presented during testing, including 52 scenes of apes engaged in natural interactions and 32 scenes of humans performing various actions (see S2 Table for details). The stimuli depicted either interactions between two animate participants (apes interacting with other apes of the same species or humans interacting with other humans; chimpanzees: 7, gorillas: 6, orangutans: 7, humans: 16) or apes or humans interacting with an inanimate object (chimpanzees: 9, gorillas: 9, orangutans: 14, humans: 16). All stimulus videos were between 2 and 10 seconds long (mean = 6.37, SD = 2.08). Animate videos with humans depicted dyadic actions such as *kick*, *pull*, *brush*, *beckon*, *scold*, or *guide*; inanimate videos with humans depicted actions such as *opening a box*, *pouring water*, *playing the drums*, *reading a book*, *fixing a bicycle*, or *putting on gloves*. We selected actions that we judged the apes to find generally familiar and avoided any actions that apes might find alarming or induce strong emotional arousal (3). A full list of stimuli shown is available in the S2 Table.

Only same-sex pairings were shown in interactions between apes or humans to avoid that the ape participants inferred potential dominance differences between males and females (4), which could affect gaze patterns. Stimuli were presented at 24 frames/second and without sound. All stimuli were edited in Lightworks (version 1.0). For each stimulus video, a mirror-flipped version was generated so that the agent could be presented on both the left and right of the screen. We counterbalanced the agent’s side for each stimulus between participants.

Footage of humans was filmed for the purpose of another study on human event cognition (1). Human actors wore colored shirts; we distributed the colors of agent and patient shirts across scenarios as evenly as possible to account for the possibility that the apes would respond to the color as a cue, rather than attending to the agent/patient dynamic (an overview of shirt color distributions is given in S2 Table).

## Apparatus and Procedure

Video stimuli were presented at a resolution of 1920*1080 pixels in the center of the 23-inch screen which was positioned above a Tobii TX300 eye tracker (for all participants but one orangutan) or a 23.8 inch screen positioned above a Tobii Spectrum eye tracker (for one orangutan; data for this participant was collected at a later date when a more optimal ape calibration option was available through the Spectrum), while the same device also recorded participants’ gaze (300 Hz sampling rate). Using Tobii Pro Lab software, the eye tracker was controlled by a Notebook Captiva NH50_70RA laptop computer, for all participants except the infants, for which a Lenova Thinkpad T15 was used.

Each trial started with an image of a piece of fruit serving as a “fixation cross”, placed at either the top or bottom of the screen (always in the same position, with presentation point counterbalanced between trials), so that participants did not already look at the position of the video stimulus when it started, since depicted actors may be visually and conceptually more salient if they happen to be looked at from the outset of stimulus presentation. Human adults, for example, are more likely to choose an event participant as the syntactic subject when already looking at the position where this event participant is located when a stimulus appears (leading to more passive sentence responses during picture description when patients are looked at first) (5–8). Ensuring that participants always fixated outside of the video stimulus boundaries guards against such inadvertent salience effects. Once participants fixated the fruit, the experimenter initiated the video stimulus, lasting between 2 and 10 seconds (see the OSF repository for example trials: https://osf.io/47wap/?view_only=8c2b20667fc441178269291fda5262bf).

**Apes.** Two types of setups were used for testing. For gorillas and chimpanzees, the eye tracker and monitor were positioned in a small enclosure (gorillas: floor: 160 * 135 * 165 * 40 cm, height: 3 m; chimpanzees: floor: 116 * 91.5 * 160 * 93.5 cm, height: 267 cm), separated from the main indoor enclosure by a sliding door and a plexiglass window (55 * 80 cm). Equipment was accessed through a separate door that opened into the keepers’ corridor (see S1 Fig A–B). At the start of each session, the door was raised and the apes could approach the testing area and sit in front of the screen. A fixed drinking nozzle was fitted in the center of the window, providing apes with access to diluted syrup. Provisioning of the syrup was controlled through a tube and catheter.

For the orangutans, the enclosure design made it difficult to implement the use of one fixed setup accessible to all participants. Instead, we implemented a height-adjustable mobile wagon (200.4±50 * 102 * 70 cm) (see S1 Fig, C) to present the eye tracker to participants through the enclosure mesh. The eye tracker was protected by a plexiglass box (63 * 102 * 70 cm) and as with the fixed setups, had a drinking nozzle on the front panel that could be accessed through the mesh. The drinking nozzle was designed to be positioned on a vertical mesh bar, so the apes’ eyes were positioned between the bars.

Sessions took place Monday-Friday in morning (09:00-12:00 hrs) and afternoon sessions (13:30-15:30 hrs). Prior to testing with the apes, we presented a series of training clips of varying stimuli (see *Supplementary text*) to ensure that they did not find certain types of video stimuli aversive, and we monitored their responses closely. During participation, apes could drink the syrup freely, and could choose whether or not to look at the screen. Overall, those apes who chose to participate in the study showed high motivation for viewing the stimuli.

Once habituated to the apparatus, the eye tracker was calibrated to the apes using a 2-point calibration procedure by displaying a short video in the corners of the screen. Calibration was recorded once, prior to all testing. Calibrations were accepted if tracking accuracy and precision were within 1° visual angle.

Each session began with a green screen for 2 seconds that indicated the start of testing. This was followed by a 4-point validation procedure to check the calibration accuracy against four fixed points on-screen. Following this, a ‘practice’ video was shown, which was not used for data collection but was designed to engage the viewer prior to the presentation of the stimuli of interest. The practice video either depicted humans or apes, so that the content of the videos was predictive of whether participants would see humans or apes in the following trials.

Trials were presented sequentially, as long as the ape kept attending to the screen and did not display any signs of distraction. All stimuli with apes as agents were presented first, followed by all stimuli with human agents. In case a participant became distracted and did not attend to a stimulus, the trial was repeated. If participants looked away in the middle of the videos, however, they were not repeated, as we aimed to test only responses to novel stimuli. Testing took place over 34 days for gorillas, 34 days for chimpanzees and 18 days for orangutans.

**Humans.** Adult participants were tested individually in a room with only the experimenter present. Participants first received written instructions to attentively watch the videos, and completed a demographic questionnaire on their linguistic background. Human adult participants also viewed all stimuli with ape agents first, followed by all stimuli with human agents. However, unlike for the ape participants, stimuli were presented in blocks (two blocks of 26 ape videos, followed by two blocks of 16 human videos). Short breaks were offered between blocks.

Human infants were tested individually in a laboratory of the research unit Developmental Psychology: Infancy and Childhood, with only an experimenter and a caregiver present. Before testing, parents completed a short questionnaire on the languages their child was exposed to, as an additional screening for any Ergative language experience. Infants were then seated on their parent’s lap in front of the eye tracker during testing, or in an infant carrier car seat, depending on which was more comfortable for them. To account for their decreased attention span, infants were presented with only half the stimuli seen by the ape and adult human participants (26 ape and 16 human videos; see S2 Table). To counter the reduction in number of trials compared with adults and apes, we increased the sample size accordingly. This included excluding any actions that might be considered unsuitable for infants, such as *hit, kick, pinch ear*.

Testing started with a short attention getter of dancing fruit followed by a 2-point calibration. Stimuli were presented in blocks (two blocks of 13 ape videos and two blocks of 8 human videos, presented in alternating order) following the calibration. Calibration was not always immediately successful, in which case to retain the infant’s attention, we continued with the presentation of the first block before attempting re-calibration. To ensure that this did not impact the overall number of human or ape stimuli viewed, the first block presented either ape or human videos first, in a counterbalanced order. If calibration was successful prior to the first block, at the start of the second block a 4-point validation was shown; otherwise, calibration was repeated. Each block started with a “practice” video, depicting either a nonhuman ape (ape blocks) or human (human block).

At the end of the session, adult participants or the infants’ caregivers were debriefed and given the chance to ask questions. For both adults and infants, sessions lasted approximately 30 minutes, including instructions and calibration.

## Data processing

In order to determine what variables influence gaze, we coded the videos for perceptual and behavioral details. This included: 1) time at which the action of the agent started, 2) whether the agent or patient moved more, 3) whether tool use was involved, 4) whether food was involved, 5) whether at any point the agent or patient was camera facing, and 6) whether at any point the agent or patient had direct gaze with the camera. For details of the coding ethogram and interrater reliability, see S3 Table.

Areas of interest for agents and patients in the videos were manually defined in the Tobii Pro Lab software (see the OSF repository for examples). The eye-tracking data were then further preprocessed in R (9). Fixations were defined using the Tobii I-VT fixation filter algorithm. For each trial, we subsumed consecutive fixations to each area of interest into gazes, linearly interpolating gaps of up to 266 ms (80 eye tracker samples) between fixations to account for saccades and short periods of potential track-loss (8). Latency (in ms) of the first gaze to either the agent or the patient was extracted. Gazes were then aggregated into time bins to reduce temporal auto-correlation (10,11). Bins were defined relative to the length of each stimulus video, so that each time bin encompassed the fixations from a segment spanning 5% of the stimulus length. This binning normalizes stimulus length and thus makes videos of different durations comparable.

## Trials were excluded from analyses if the first gaze into a stimulus occurred later than 1000 ms after video onset or if no fixations were detected. Trials from apes were removed if they looked at the area of the video before it started playing on screen (as observed by the experimenter). For the infants that were not fully excluded, trials were excluded due to poor or missing calibrations or because the infants were not attending to the stimuli. Trials in which participants never fixated on the agent or patient or in which none or less than 50 ms of eye tracker samples were registered before the first fixation was detected were also excluded. In addition, three video stimuli presented to human adults, and two video stimuli presented to apes and human adults, were excluded from analyses due to technical errors with the videos; one video stimulus presented to apes and human adults was also excluded, because the agent and patient were considered too ambiguous. Overall, 3537 (out of 5628 total trials) trials were included in the statistical analyses.

## Statistical analyses

The data were analyzed in R (version 4.2.1). We ran Bayesian multilevel models on the single trial level using the RStan package (9,12), which fits Bayesian models using a Hamiltonian Markov chain Monte Carlo algorithm. Markov chain convergence was assessed using standard diagnostics (number of effective samples, the Gelman-Rubin diagnostic, and visual inspection of trace plots).

Our outcome variable was the AOI (e.g., Agent, Patient, or Other). To adjust for potential confounding factors, the model included as predictors: the ratio between the mean agent and the mean patient AOI size between the video stimulus start and the onset of the first gaze (S5 Fig); the category of the depicted event (“social” events with both an ape or human agent and patient or events where an ape or human agent interacted with an inanimate object, or with food); difference in movement between agent and patient (coding which moved more; S6 Fig); the participants’ species and the depicted species, as well as group-level predictors for the individual stimuli and participants. In most stimuli, the action of interest started immediately at the onset of video presentation. In 25 stimuli, however, the action started only after video onset (on average 0.24 seconds after); for these stimuli, the time points before the action started were excluded from the models.

*Trial* *averaged model comparison*

We first fit two models designed to test whether there was an overall difference in the frequency of Agents vs Patients gazes, averaging within trials. To do this, we calculated the trial-level proportion of gazes to each AOI, using a Dirichlet distribution for our response variable (the proportion of gazes directed to Agent, Patient, and Other over the entire trial, where the three components sum to 1).

$$y \sim Dirichlet(\mu\Phi)$$

$$\mu=softmax(\eta)$$

Where **y** is a vector containing the trial level proportions of gazes at each AOI, **α** is the parameter vector of the Dirichlet distribution, which we re-parameterize in terms of mean vector **μ** and dispersion parameter φ. We specify linear models for each element in **η**, which represent the latent-scale expected proportion of gazes for each AOI.


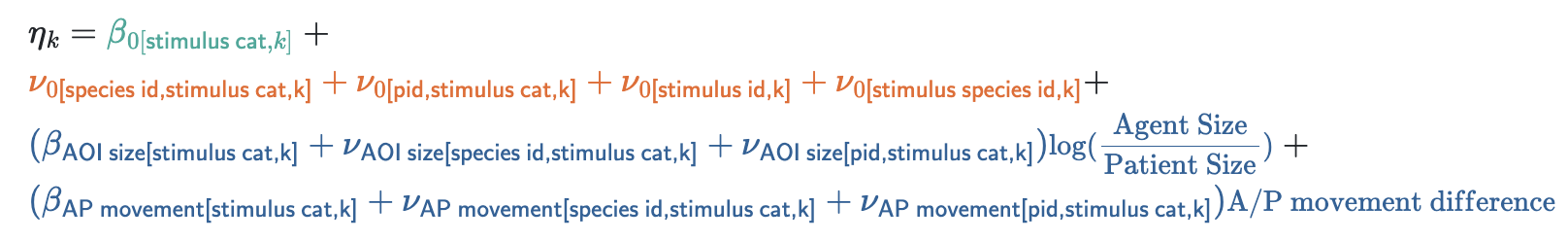


We set weakly-regularizing priors on all model parameters:

$$\beta\sim Normal(0,1)$$

$$\sigma\sim Exponential(1)$$

$$\phi\sim Gamma(0.1, 0.1)$$

To set up our null model (equal gazes to Agents and Patients, conditional on covariates), we used the same model structure as just described, except that we forced the linear model for the Agent AOI to be equal to the Patient AOI rather than estimate each of them. This forces the two AOIs to always have the same conditional probability, which is what we would expect if Agents and Patients were exchangeable.

After fitting both the null and alternative models, we compared them using approximate leave-one-out cross-validation via the expected pointwise predictive density (ELPD). The ELPD is approximated by Pareto-smoothed importance sampling (PSIS) of the posterior (13). To assess relative model performance, we used model stacking, which allocates weights to models such that they jointly maximize predictive accuracy (14). These stacking weights are interpreted as the probability that a model will perform better than any other model in the comparison set (in this case, just the null and alternative models). ELPD and stacking weights were computed using the 'loo' R package (15).

*Time series model*

We next ran Bayesian multilevel models on the level of each time point in a trial. We used a categorical distribution for our outcome variable, the AOI category (e.g., Agent, Patient, or Other), that a participant was gazing at during time $t$, where $t\in\left[ 1,20 \right]$ indexes the 5% interval of the total duration of a trial such that $t=1$ corresponds to the time interval $[0,0.05)$, $t=2$ corresponds to $[0.05,0.10)$, etc. In principle, a participant could gaze at multiple AOIs during an interval. However, given that these intervals correspond to approximately 300 ms (mean = 319.939, sd = 103.783; varies by stimulus), and the average duration of eye fixations in humans is 200-300 ms (16), a time bin should usually capture only 1 unique fixation event. As such, heterogeneity within such a short time interval may be best understood as measurement error.

That said, the choice of a 5% window is somewhat arbitrary. Ideally, we would choose a binning window length that is short enough not to average over true fixations, while not being so short that adjacent samples are perfectly auto-correlated, a ubiquitous problem in eye- tracking research and time series modeling more generally. We can quantify the information gained by decreasing interval length by looking at the correlation between the data at 5% intervals vs 2.5% intervals–a doubling of temporal resolution. We found that, averaging over the three AOIs, the correlation between the 2.5% and 5% binned variables was $\rho=0.929$. This suggested that little information would be gained by further increases in temporal resolution, and thus we used 5% intervals to balance data loss with computational burden.

A key feature in our study was to include inanimate objects in semantic roles, which complicated matters. For example, if an animate agent grasps an inanimate object, the gaze is likely to fall on the agent's hand but also on the patient (e.g. an apple). This results in another source of heterogeneity within time intervals (i.e., a mixture of gazes between different AOIs), that is, for some videos, the Agent and Patient AOIs overlap on the screen, such that it is possible for a participant to be gazing at both simultaneously. Thus, even if there were no measurement error we would sometimes observe a mixture of AOI gazes during a short time window. To propagate uncertainty in our model due to both measurement error and overlapping AOIs on the screen, we weight the probability of gazing at each AOI (a latent state, denoted by $K$, where $k = 1$ indicates Agent, $k = 2$ indicates Patient, and $k = 3$ indicates Other) by the observed proportion of gazes for each AOI during an interval $t$, denoted $Y$ where $y_{t,k = 1}$ indicates the proportion of agent gazes during $t$, $y_{\left[ t,k = 2 \right]}$ indicates the proportion of patient gazes, and $y_{\left[ t,k = 3 \right]}$ indicates the proportion of other gazes.


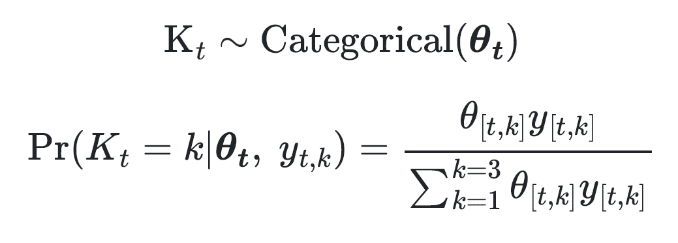


Where $\theta_{t}$ is a vector of probabilities for each latent AOI state, (e.g., $\theta_{\left[ t,k = 1 \right]}$ is the model probability of Agent during time $t$ such that $\theta_{t}$ sums to 1. With this approach, we were able to model AOI gaze as a latent categorical variable while propagating the uncertainty that is inherent to the data generating process (i.e., measurement error).

We can then make $\theta_{t}$ a function of predictors in a generalized linear model using the softmax link function. As such, there were $max\left( K \right)-1$ linear models for the latent “scores”, denoted $s$, which the softmax function maps to the vector of probabilities $\theta_{t}$. “Other” was set as the reference category, so that $s_{\left[ t,k=3 \right]}=0$ for all $t$.

Below we describe the linear model, first as a simplified version using natural language, and then a formal definition:

**Natural language definition**


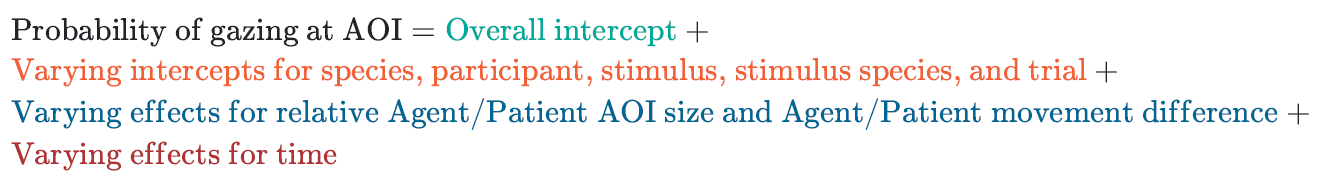


**Formal definition**

**
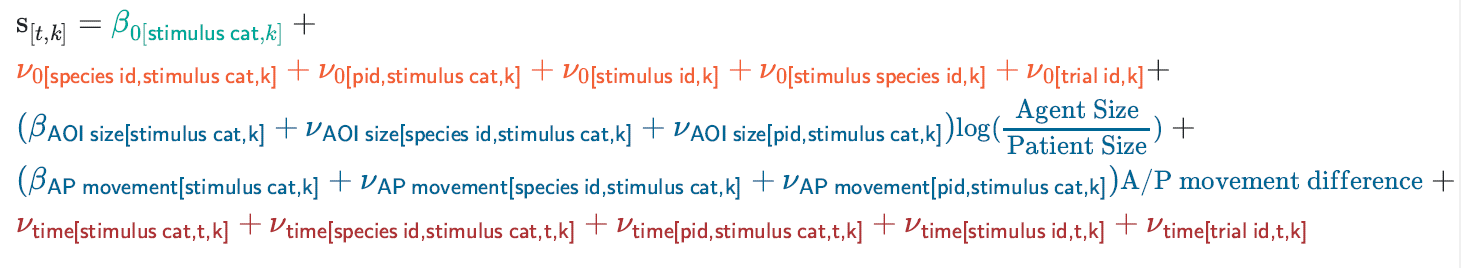
**

Where “stimulus cat” is an index of whether the video (stimulus) being viewed is inanimate (food), inanimate (non-food), or social. Stimulus ID indexes the identity of the specific video being viewed in a trial, while Stimulus species ID indexes the identity of the species featured in a video. $\beta$ indicates an overall (also known as “fixed”) effect, while $\nu$ indicates a varying (also known as “random” or “mixed”) effect. The varying effects for time co-vary according to a Gaussian process with an exponential kernel:


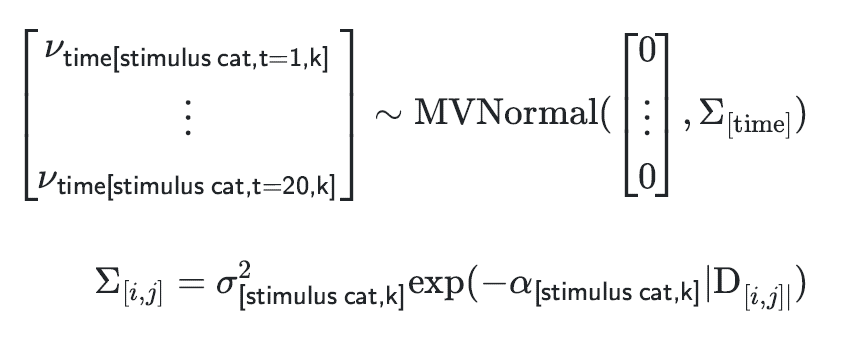


Where $D$ is a matrix of pairwise distances between time intervals. Adjacent intervals have a distance of 0.05 (corresponding to 5% time bins), intervals two steps apart have a distance of 0.1, and so on. The relative “wiggliness” or rigidity of these time functions are controlled by the parameter $\alpha$ (large values of $\alpha$ imply relatively wiggly functions that change rapidly, small values imply relatively rigid functions that change slowly). The magnitude of the functions (i.e., the extent to which they affect the latent scores) is controlled by $\sigma^{2}$.

The time varying effects for species, participant (pid), stimulus, and trial are modeled using hierarchical Gaussian processes, which means rather than estimate each time function for each group independently, the model estimates shared $\alpha$ and $\sigma^{2}$ parameters for each level. Thus, each species, participant, stimulus, and trial can have a unique time smooth, but these functions are regularized via partial pooling to reduce over fitting. As an additional layer of regularization given the high number of parameters in our model, we employed a Dirichlet decomposition for each set of varying effects (17,18). This means that we parameterized the variance of each vector of varying effects $\nu$ in terms of the total variance for that set (e.g., the total variance of the intercepts, or time effects) and a simplex $\phi$ consisting of level-specific variance components. For example, if $\sigma^{2}$ = 2, and $\phi_{pid}=0.3$, then the variance for the participant-level effects is 2(0.3) = 0.6. By avoiding separate variance parameters for each level (i.e., separate $\sigma^{2}$ priors for species, participants, stimuli, trials), we constrain the prior predictive variance.

We set generic, weakly-regularizing priors on all model parameters:

$$\beta\sim Normal(0,1)$$

$$\sigma^{2}\sim Exponential(1)$$

$$\alpha\sim Normal(0, 10)$$

$$\phi\sim Dirichlet(2)$$

See Stan code on the SOF repository for additional details on the implementation of this model.

**Supplementary text**

**Ape testing**

*Habituation procedure*

Initial habituation to the eye tracker took part in two phases. In the first phase, apes were shown the liquid reward to encourage them to approach and inspect the screen. If they attended to the screen then a video would be played. Stimuli were presented using either Tobii Studio (for gorillas) or Tobii Pro Lab (for chimpanzees and orangutans). In the first phase, 75 videos depicting varied species, actions and interactions were played, to engage participants, and to determine interest and emotional response to different types of videos. The goal was to ensure that participants were comfortable to approach the screen and attend to the videos, and that they kept a good position during viewing. After successfully viewing all footage of the first phase, they were then shown footage from phase 2. This consisted of 16 videos depicting a variety of species, selected for their causal structure. We used gaze responses to this footage to help determine AOI regions for the test footage, and to finalize the most appropriate footage for testing. Habituation and calibration took place over two months for the gorillas, three months for the chimpanzees, and four months for the orangutans.

**Test footage collation**

Footage of apes were collated from a combination of captive and wild footage from our own archives, other researchers and the Disney movie Chimpanzee, as well as filmed for the purposes of this study at Zurich Zoo (see S2 Table for full list). Natural scenes depicted footage of dyadic social interactions such as play, grooming, approaching and infant suckling, as well as inanimate interactions such as foraging, eating, using enrichment and tool use. We chose stimuli of relatively neutral content so as to avoid arousal responses which could distract participants.

**Variable coding scheme**

The coding scheme is detailed in S3 Table. To determine interrater reliability for these variables, coding was conducted by two coders. For the variables time action starts and agent-patient movement difference, both coders coded and discussed differences for 20 video clips; they then independently coded an additional 21 test clips (25%). For the variables direct gaze at camera and orientation towards camera, due to the low number of instances of each behavior, both coders coded all videos. Interrater reliability was calculated using Cohen's kappa. Values are reported next to each variable in the table.

**References**

1. Isasi-Isasmendi A, Andrews C, Flecken M, Laka I, Meyer M, Bickel B, et al. The agent preference in visual event apprehension. Open Mind. 2023;7:240–82.

2. Segal SC, Marquis AR, Moulson MC. Are our samples representative? Understanding whether temperament influences infant dropout rates at 3 and 7 months. Infant Behavior and Development. 2021;65:101630.

3. Kano F, Hirata S, Deschner T, Behringer V, Call J. Nasal temperature drop in response to a playback of conspecific fights in chimpanzees: A thermo-imaging study. Physiology and Behavior. 2016;155:83–94.

4. Lewis LS, Kano F, Stevens JMG, DuBois JG, Call J, Krupenye C. Bonobos and chimpanzees preferentially attend to familiar members of the dominant sex. Animal Behaviour. 2021;177:193–206.

5. Esaulova Y, Dolscheid S, Reuters S, Penke M. The alignment of agent-first preferences with visual event representations: contrasting German and Arabic. Journal of Psycholinguistic Research. 2021;(0123456789).

6. Gleitman LR, January D, Nappa R, Trueswell JC. On the give and take between event apprehension and utterance formulation. Journal of Memory and Language. 2007;57(4):544–69.

7. Myachykov A, Garrod S, Scheepers C. Determinants of structural choice in visually situated sentence production. Acta Psychologica. 2012;141(3):304–15.

8. Pokhoday M, Shtyrov Y, Myachykov A. Effects of visual priming and event orientation on word order choice in russian sentence production. Front Psychol. 2019;10:1661.

9. R Core Team. R: A language and environment for statistical computing. Vienna: R Foundation for Statistical Computing; 2022.

10. Barr DJ. Analyzing ‘visual world’ eyetracking data using multilevel logistic regression. Journal of Memory and Language. 2008;59(4):457–74.

11. Cho SJ, Brown-Schmidt S, Lee W yeol. Autoregressive generalized linear mixed effect models with crossed random effects: an application to intensive binary time series eye-tracking data. Psychometrika. 2018;83(3):751–71.

12. Stan Development Team. Stan Modeling Language Users’ Guide and Reference Manual [Internet]. 2023. Available from: https://mc-stan.org

13. Vehtari A, Gelman A, Gabry J. Practical Bayesian model evaluation using leave-one-out cross-validation and WAIC. Stat Comput. 2017;27(5):1413–32.

14. Yao Y, Vehtari A, Simpson D, Gelman A. Using stacking to average Bayesian predictive distributions. Bayesian Analysis. 2018;10:10.1214/17-BA1091.

15. Vehtari A, Gelman A, Gabry J, Yao Y. Package ‘loo’. Efficient leave-one-out cross-validation and WAIC for Bayesian models. 2021.

16. Salthouse TA, Ellis CL. Determinants of eye-fixation duration. AJP. 1980;93(2):207–34.

17. Zhang YD, Naughton BP, Bondell HD, Reich BJ. Bayesian regression using a prior on the model fit: The R2-D2 shrinkage prior. Journal of the American Statistical Association. 2022;117(538):862–74.

18. Aguilar JE, Bürkner PC. Intuitive joint priors for Bayesian linear multilevel models: The R2D2M2 prior. Electron J Statist. 2023;17(1).
