# Supplementary material for: Humans and great apes visually track event roles in similar ways
Source: PLoS Biol. 2024 Nov 26;22(11):e3002857. doi: 10.1371/journal.pbio.3002857 (PMC11593759; doi:10.1371/journal.pbio.3002857)
Supplement: S1 Fig — (DOCX) [file pbio.3002857.s002.docx]

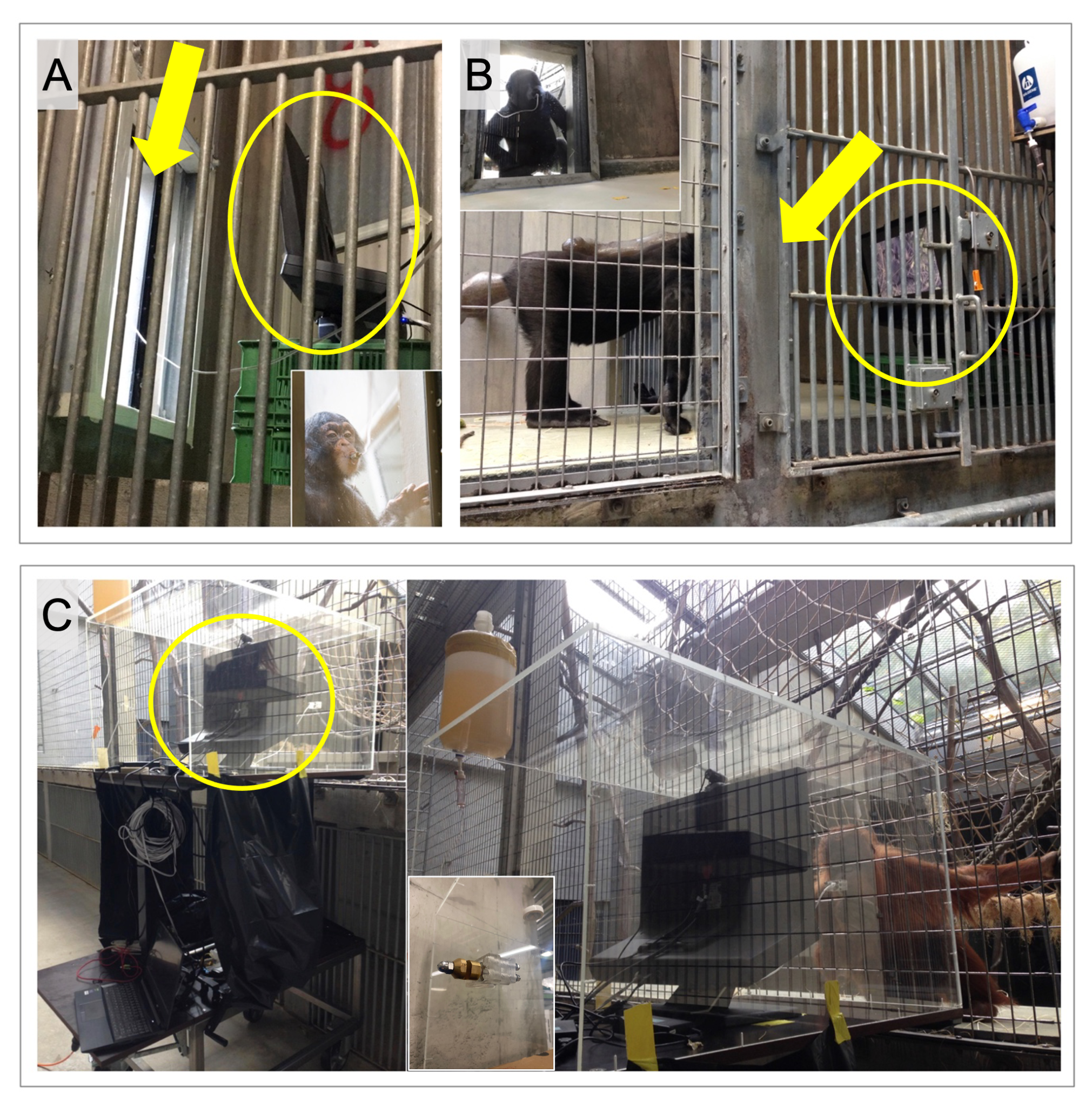


S1 Fig. Eye-tracking setups at Basel Zoo. A: fixed setup for chimpanzees. B fixed setup for gorillas. C: mobile setup for orangutans; indent shows drinking nozzle with hook to attach to mesh. Circles indicate display monitor and arrows indicate viewing window.
