# Supplementary material for: Humans and great apes visually track event roles in similar ways
Source: PLoS Biol. 2024 Nov 26;22(11):e3002857. doi: 10.1371/journal.pbio.3002857 (PMC11593759; doi:10.1371/journal.pbio.3002857)
Supplement: S2 Fig — (DOCX) [file pbio.3002857.s003.docx]

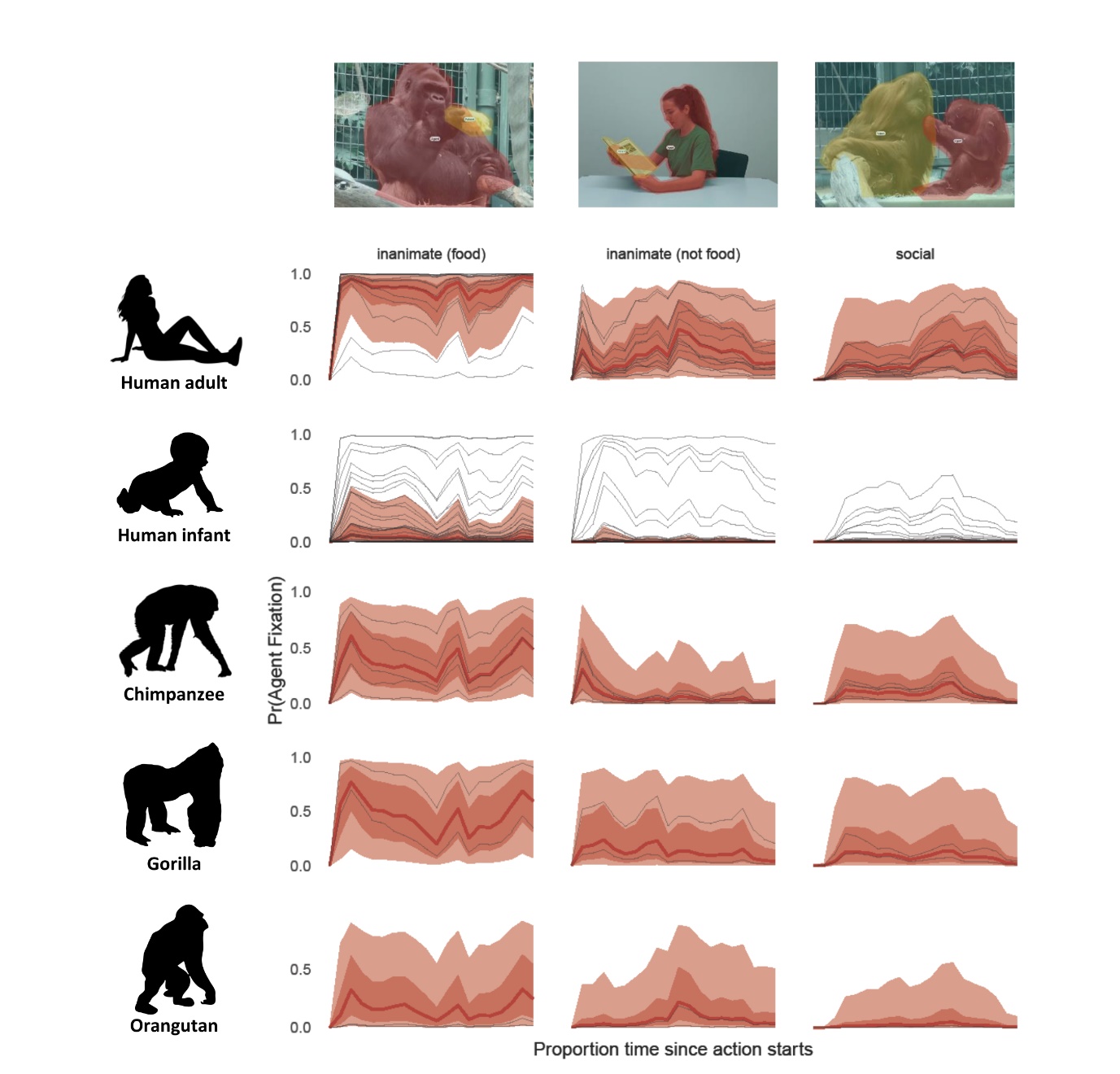


S2 Fig: Time course of gazes towards agent in the videos, predicted with a categorical Bayesian multilevel model. Thick lines represent the grand mean, thin lines represent individual participants. Time point 0 on the x-axis indicates action start time, normalized across stimuli; fixations were aggregated into 5% time bins; shaded ribbons indicate 50 and 90% credible intervals, respectively.
