# Supplementary material for: Humans and great apes visually track event roles in similar ways
Source: PLoS Biol. 2024 Nov 26;22(11):e3002857. doi: 10.1371/journal.pbio.3002857 (PMC11593759; doi:10.1371/journal.pbio.3002857)
Supplement: S5 Fig — (DOCX) [file pbio.3002857.s006.docx]

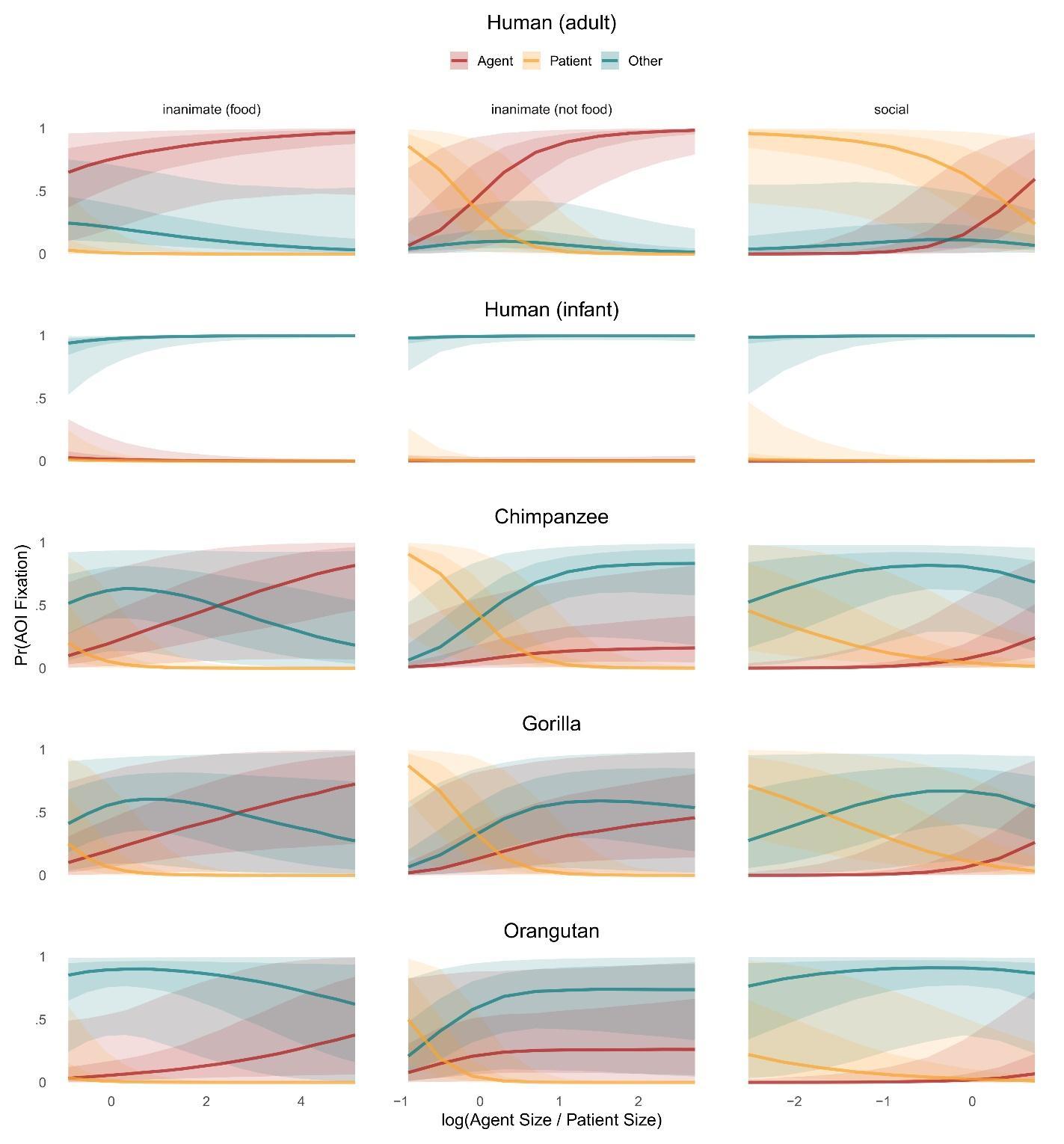

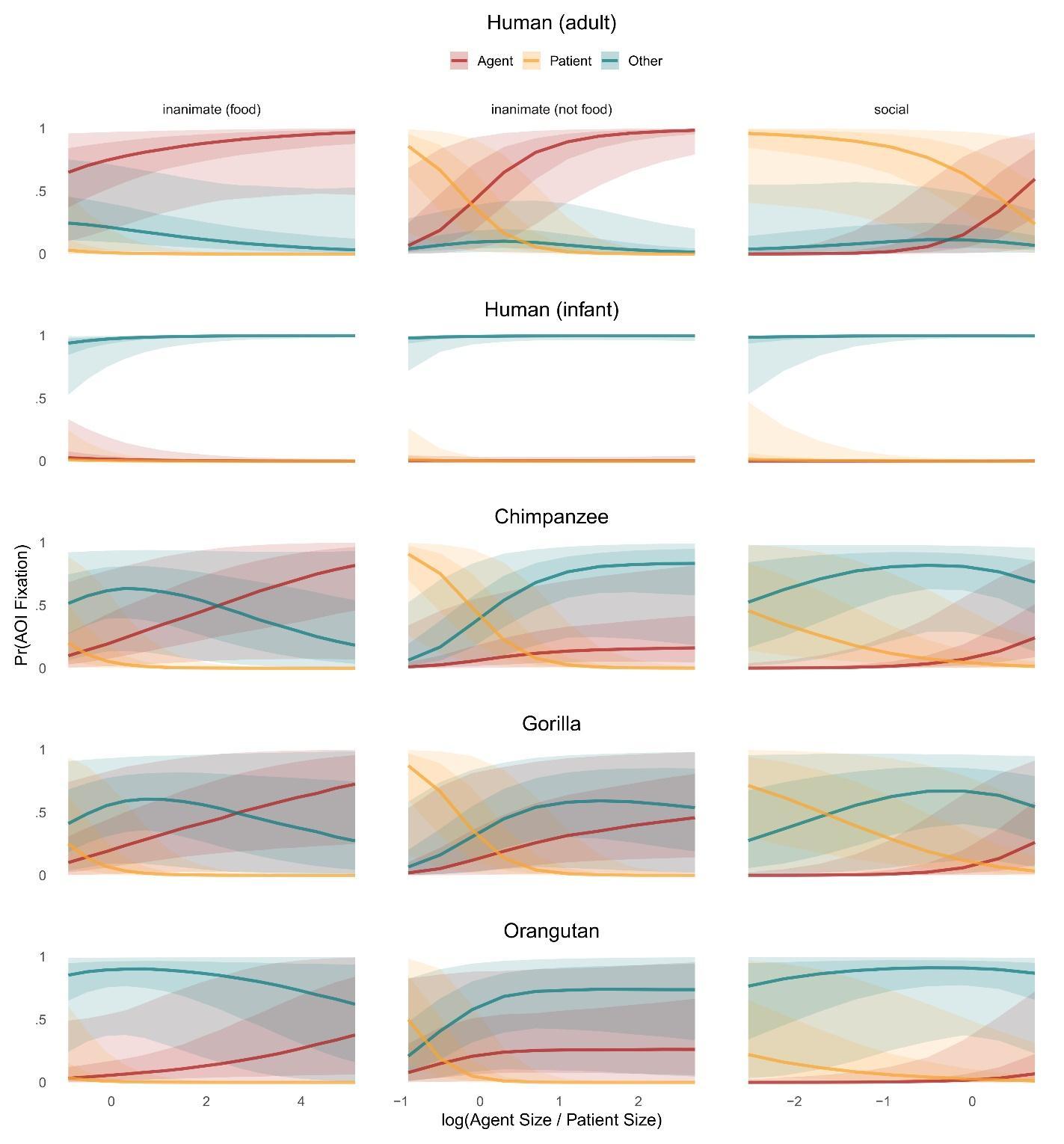

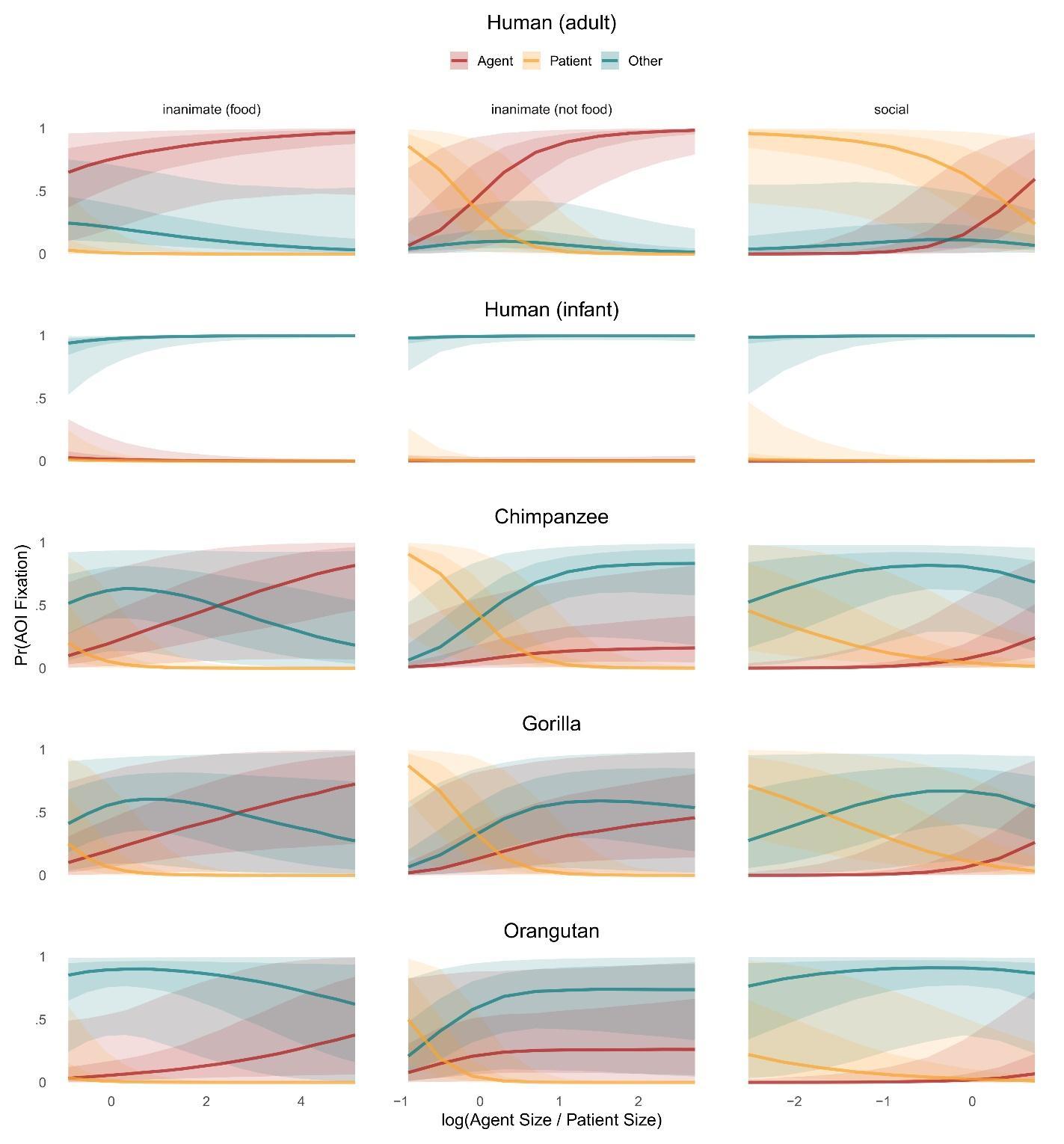


S5 Fig. AOI fixation probability as a function of AOI size. AOIs are depicted by color: agent (red), patient (orange), and other information (turquoise).
