# Supplementary material for: Humans and great apes visually track event roles in similar ways
Source: PLoS Biol. 2024 Nov 26;22(11):e3002857. doi: 10.1371/journal.pbio.3002857 (PMC11593759; doi:10.1371/journal.pbio.3002857)
Supplement: S6 Fig — (DOCX) [file pbio.3002857.s007.docx]

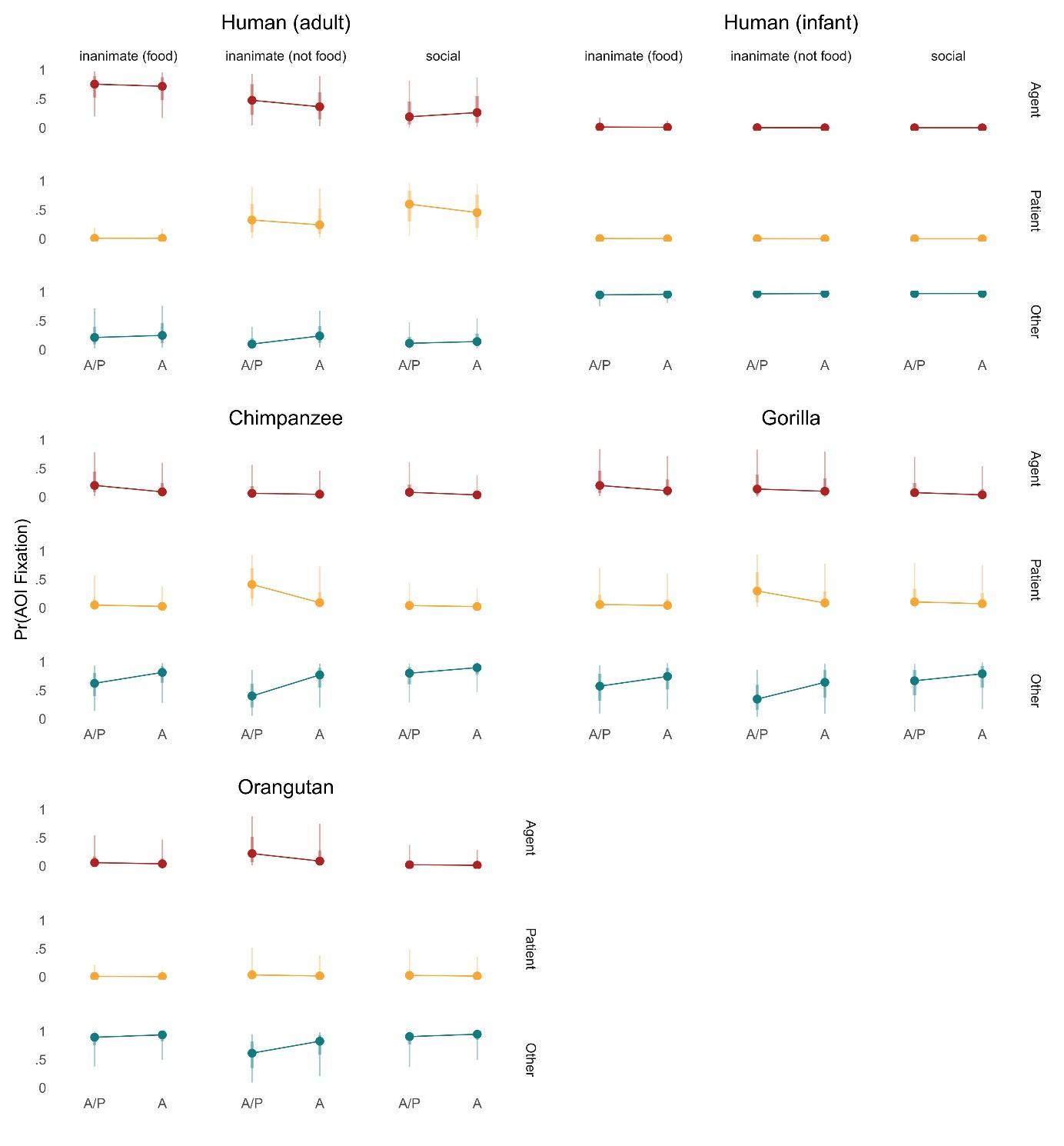
S6 Fig. AOI fixation probability by movement difference between agent and patient. A = agent moves more than patient. A/P = agent and patient move a similar amount. Colors depict the agent (red), patient (orange), and other information (turquoise). Individual numerical values underlying this figure are available in the file fig_S6_values.csv on the OSF repository.
