# Supplementary material for: Humans and great apes visually track event roles in similar ways
Source: PLoS Biol. 2024 Nov 26;22(11):e3002857. doi: 10.1371/journal.pbio.3002857 (PMC11593759; doi:10.1371/journal.pbio.3002857)
Supplement: S7 Fig — (DOCX) [file pbio.3002857.s008.docx]

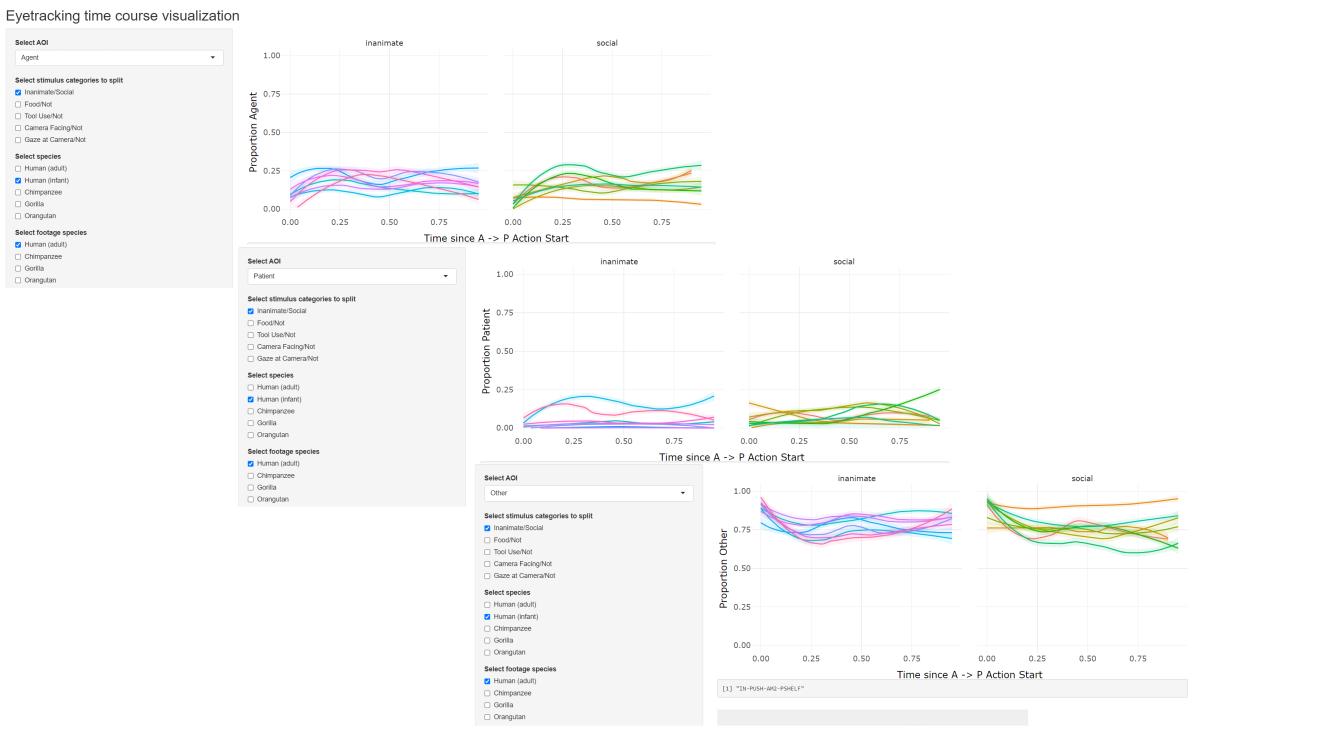


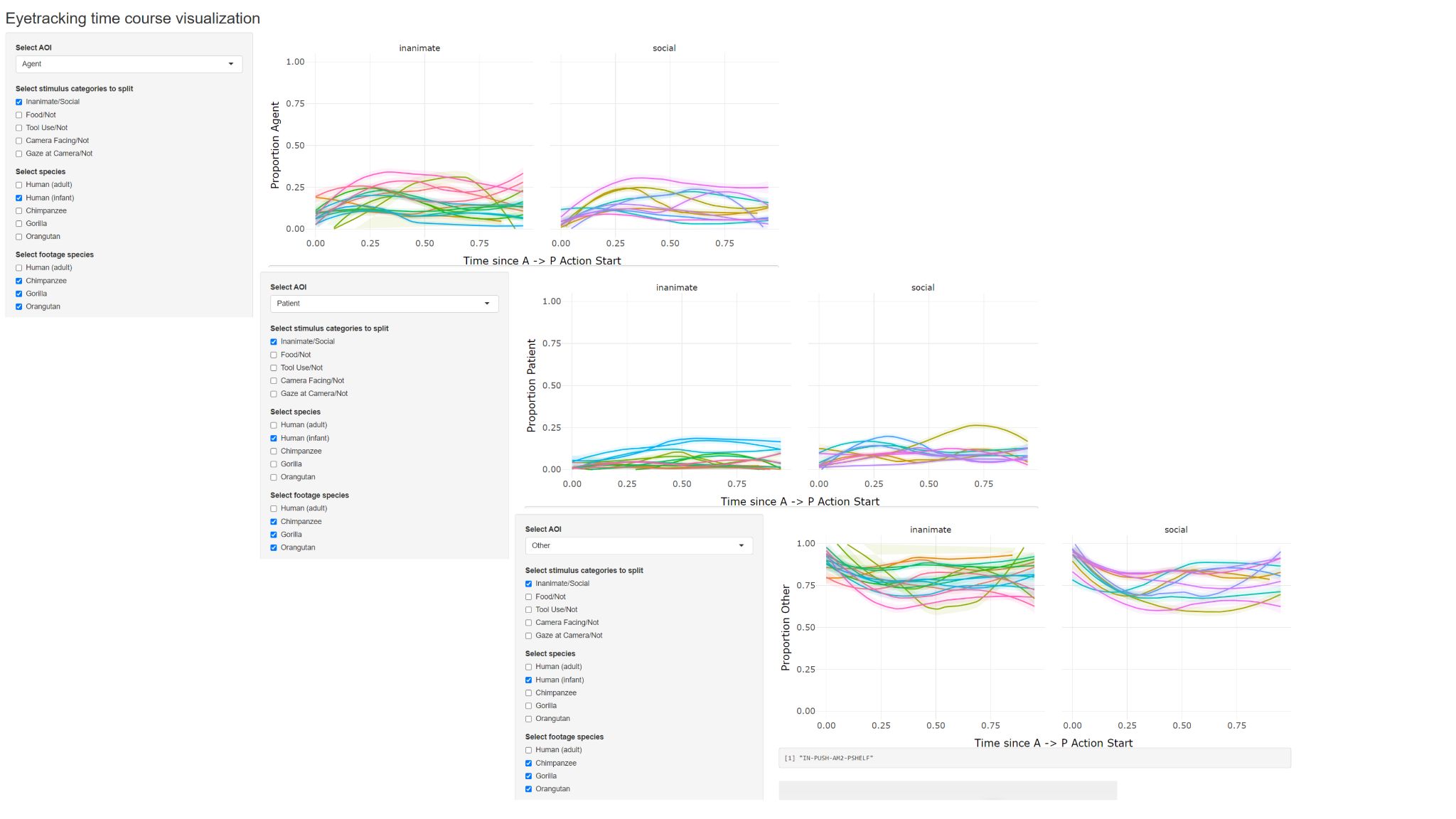


S7 Fig. Gaze for infant participants when viewing human-only footage (top panel) and ape-only footage (bottom panel) to agent (left), patient (middle) and other (right). Figures are extracted from the shiny app dashboard on the OSF, which depicts the time course of gaze by stimulus species. The dashboard can also be accessed at: https://dataplatform.evolvinglanguage.ch/eventcog_eyetracking
