# Supplementary material for: Humans and great apes visually track event roles in similar ways
Source: PLoS Biol. 2024 Nov 26;22(11):e3002857. doi: 10.1371/journal.pbio.3002857 (PMC11593759; doi:10.1371/journal.pbio.3002857)
Supplement: S8 Fig — (DOCX) [file pbio.3002857.s009.docx]

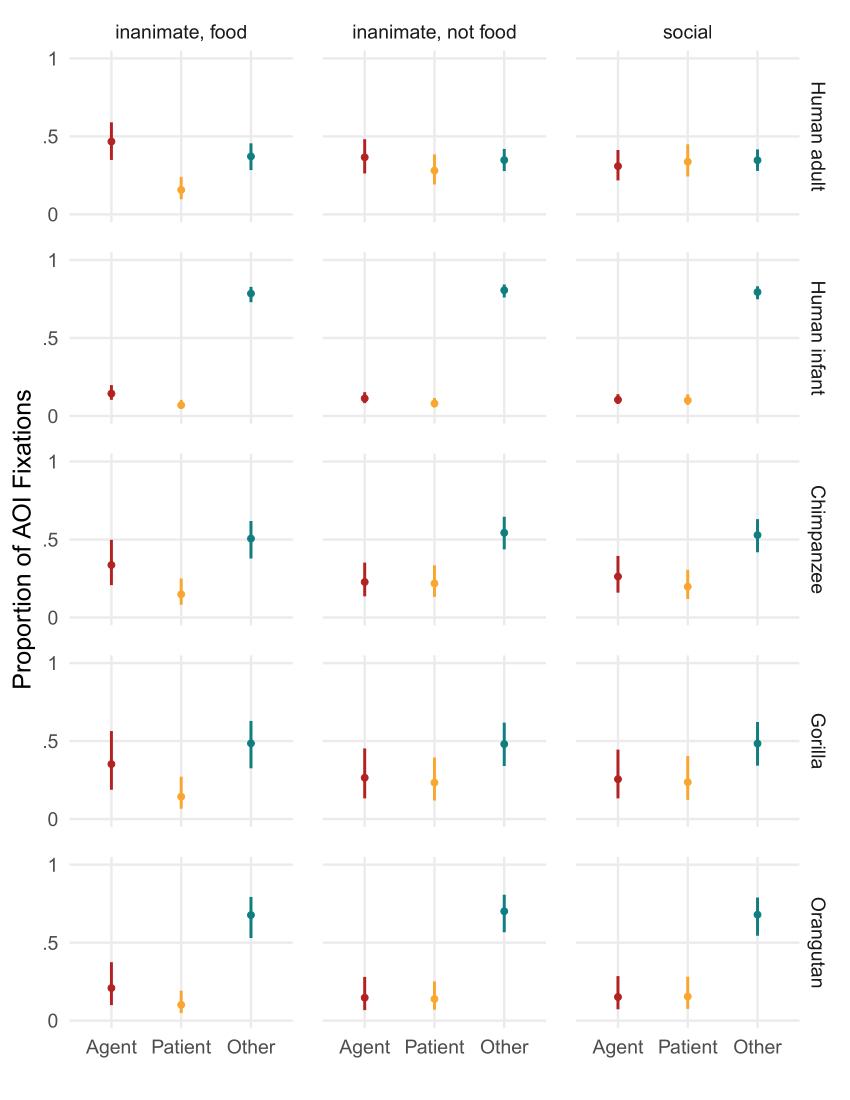


S8 Fig. Proportion of fixations to each area of interest, aggregated across time points. Individual numerical values underlying this figure are available in the file fig_S8_values.csv on the OSF repository.
