# Supplementary material for: Humans and great apes visually track event roles in similar ways
Source: PLoS Biol. 2024 Nov 26;22(11):e3002857. doi: 10.1371/journal.pbio.3002857 (PMC11593759; doi:10.1371/journal.pbio.3002857)
Supplement: S9 Fig — (DOCX) [file pbio.3002857.s010.docx]

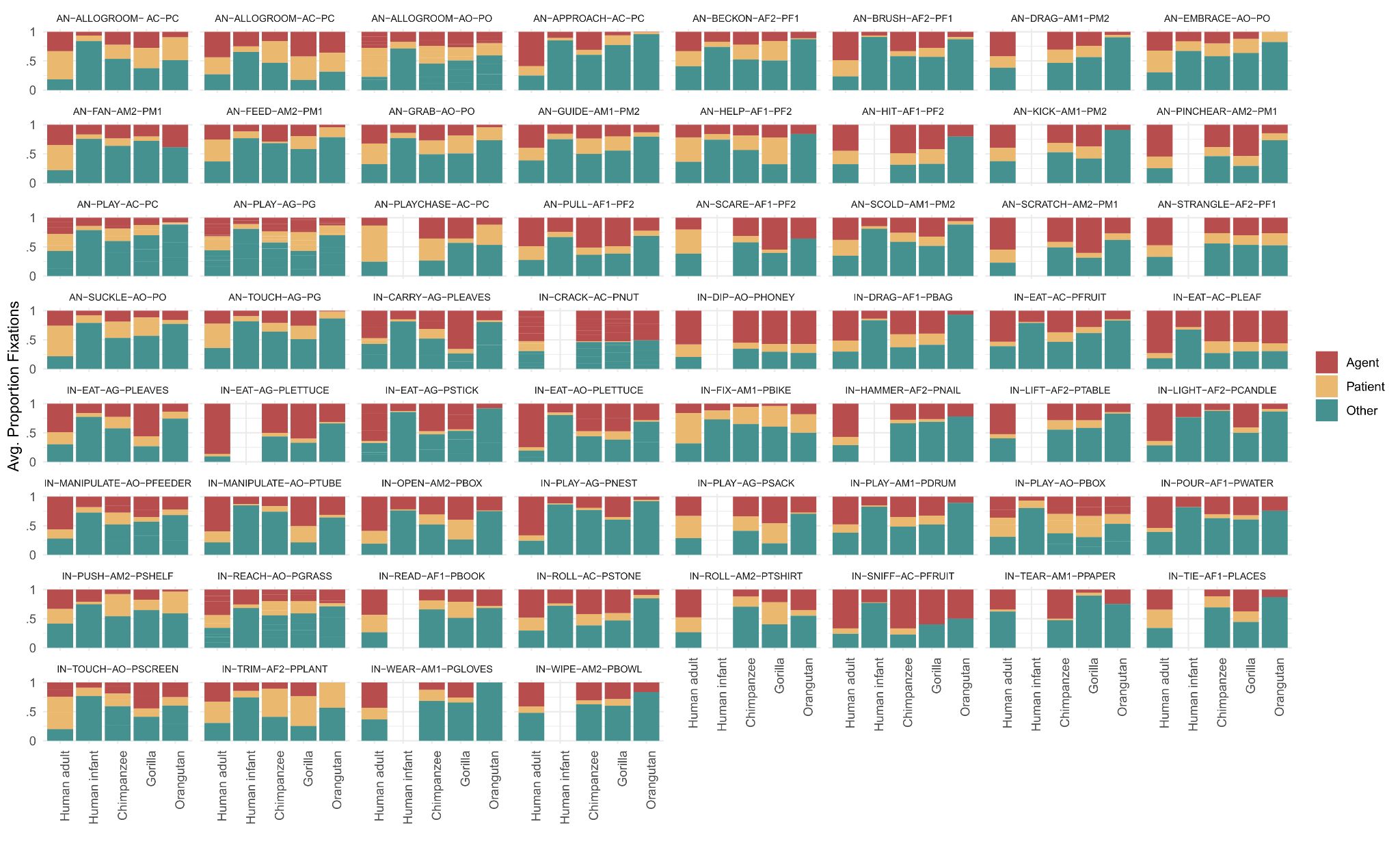


S9 Fig. Average proportion of fixations for each species x stimulus, averaging over time, trial and participant. Action verbs are listed after AN/IN- in each sequence name. AN = animate scene, IN = inanimate scene, A = agent, F = female, M = male, P = patient, C = chimpanzee, G = gorilla, O = orangutan.
