# Supplementary material for: Humans and great apes visually track event roles in similar ways
Source: PLoS Biol. 2024 Nov 26;22(11):e3002857. doi: 10.1371/journal.pbio.3002857 (PMC11593759; doi:10.1371/journal.pbio.3002857)
Supplement: S1 Table — (DOCX) [file pbio.3002857.s011.docx]

S1 Table. Model comparison differentiating event role specification as a predictor of agent-patient gaze proportion

| **Human adults** | | | | | |
| --- | --- | --- | --- | --- | --- |
|  | ELPD_LOO_ | SE(ELPD_LOO_) | Δ_ELPD_ | SE(Δ_ELPD_) | weight |
| *Null model* | 1047.6 | 39.0 | -509.9 | 43.5 | 0.058 |
| *Alternative model* | 1557.5 | 46.2 |  |  | 0.942 |
| **Human infants** | | | | | |
|  | ELPD_LOO_ | SE(ELPD_LOO_) | Δ_ELPD_ | SE(Δ_ELPD_) | weight |
| *Null model* | 8762.4 | 184.4 | -191 | 22.3 | 0.086 |
| *Alternative model* | 8953.3 | 181.4 |  |  | 0.914 |
| **Apes** | | | | | |
|  | ELPD_LOO_ | SE(ELPD_LOO_) | Δ_ELPD_ | SE(Δ_ELPD_) | weight |
| *Null model* | 1325.9 | 63.8 | -167 | 23.2 | 0.086 |
| *Alternative model* | 1492.9 | 65.6 |  |  | 0.914 |

Note. *Null model* = without event role as predictor. *Alternative model* = with event role as predictor. ELPD_LOO_ is the expected log pointwise density, which indicates the predictive accuracy of the model. Δ_ELPD_ indicates the difference in predictive accuracy between the null model and the alternative model. Model weights are stacking weights, indicating the probability that the alternative model will predict new data better than the null model.
