# Supplementary material for: Humans and great apes visually track event roles in similar ways
Source: PLoS Biol. 2024 Nov 26;22(11):e3002857. doi: 10.1371/journal.pbio.3002857 (PMC11593759; doi:10.1371/journal.pbio.3002857)
Supplement: S2 Table — (DOCX) [file pbio.3002857.s012.docx]

S2 Table. Full list of video footage presented

| **Description** | **Species** | **Sex** | **Category** | **Infants viewed** | **Agent Shirt color** | **Patient shirt color** |
| --- | --- | --- | --- | --- | --- | --- |
| Beckon/call over | Human | F to F | Social | Y | Purple | Orange |
| Brush | Human | F to F | Social | Y | Yellow | White |
| Drag | Human | M to M | Social |  | Green | Red |
| Fan | Human | M to M | Social | Y | White | Black |
| Feed | Human | M to M | Social | Y | White | Orange |
| Guide | Human | M to M | Social | Y | Black | Orange |
| Help get up | Human | F to F | Social | Y | Turquoise | Black |
| Hit | Human | F to F | Social |  | Green | Orange |
| Kick | Human | M to M | Social |  | White | Yellow |
| Pinch ear | Human | M to M | Social |  | Turquoise | White |
| Pull | Human | F to F | Social | Y | Red | Purple |
| Scare | Human | F to F | Social |  | Black | Red |
| Scold | Human | M to M | Social | Y | Yellow | Black |
| Scratch | Human | M to M | Social |  | Yellow | Purple |
| Strangle | Human | F to F | Social |  | White | Red |
| Tread on | Human | F to F | Social |  | Green | Blue |
| Drag bag | Human | F | Inanimate | Y | Yellow |  |
| Fix bike | Human | M | Inanimate | Y | Orange |  |
| Hammer | Human | F | Inanimate |  | Black |  |
| Lift table | Human | F | Inanimate |  | Red |  |
| Light candle | Human | F | Inanimate | Y | Green |  |
| Open box | Human | M | Inanimate | Y | Blue |  |
| Play drum | Human | M | Inanimate | Y | White |  |
| Pour water | Human | F | Inanimate | Y | Orange |  |
| Push shelf | Human | M | Inanimate | Y | Orange |  |
| Read a book | Human | F | Inanimate |  | Green |  |
| Roll | Human | M | Inanimate |  | Purple |  |
| Tear paper | Human | M | Inanimate |  | Purple |  |
| Tie shoe laces | Human | F | Inanimate |  | Purple |  |
| Trim plant | Human | F | Inanimate | Y | Yellow |  |
| Put on glove | Human | M | Inanimate |  | Black |  |
| Wipe bowl | Human | M | Inanimate |  | Yellow |  |
| Eat fruit | Chimpanzee | unknown | Inanimate food | Y |  |  |
| Nut cracking | Chimpanzee | juvenile M | Inanimate food |  |  |  |
| Play chase | Chimpanzee | infants | Social |  |  |  |
| Play with tube | Orangutan | F | Inanimate | Y |  |  |
| Play with part of nest | Gorilla | F | Inanimate | Y |  |  |
| Play with sack | Gorilla | F | Inanimate |  |  |  |
| Touch | Gorilla | F to F | Social | Y |  |  |
| Allogroom | Orangutan | F to F | Social |  |  |  |
| Allogroom | Orangutan | F to F | Social |  |  |  |
| Play | Orangutan | F to F | Social | Y |  |  |
| Approach and embrace | Orangutan | F to F | Social | Y |  |  |
| Nut cracking | Chimpanzee | F | Inanimate food |  |  | Y |
| Reach for grass | Orangutan | F | Inanimate food |  |  |  |
| Reach for grass | Orangutan | F | Inanimate food |  |  |  |
| Reach for grass | Orangutan | F | Inanimate food |  |  |  |
| Reach for grass | Orangutan | F | Inanimate food | Y |  |  |
| Play | Gorilla | F to F | Social |  |  |  |
| Eat banana leaves | Gorilla | F | Inanimate food | Y |  |  |
| Walk with leaves | Gorilla | F | Inanimate food | Y |  |  |
| Walk with leaves | Gorilla | F | Inanimate food |  |  |  |
| Eat stick | Gorilla | M | Inanimate food | Y |  |  |
| Eat stick | Gorilla | M | Inanimate food |  |  |  |
| Nut cracking | Chimpanzee | infant | Inanimate food |  |  | Y |
| Eat stick | Gorilla | M | Inanimate food |  |  |  |
| Eat | Gorilla | M | Inanimate food |  |  |  |
| Eat | Orangutan | F | Inanimate food |  |  |  |
| Eat | Orangutan | F | Inanimate food | Y |  |  |
| Play | Gorilla | F to F | Inanimate | Y |  |  |
| Bite/allogroom | Gorilla | F to F | Social |  |  |  |
| Allogroom | Orangutan | F to F | Social | Y |  |  |
| Allogroom | Orangutan | F to F | Social |  |  |  |
| Suckle | Orangutan | F to F | Social | Y |  |  |
| Honey dipping | Orangutan | M | Inanimate food |  |  | Y |
| Nut cracking | Chimpanzee | infant | Inanimate food |  |  | Y |
| Using touchscreen | Orangutan | M | Inanimate |  |  |  |
| Taking food from feeder | Orangutan | M | Inanimate food |  |  |  |
| Taking food from feeder | Orangutan | M | Inanimate food | Y |  |  |
| Using touchscreen | Orangutan | M | Inanimate | Y |  |  |
| Play | Orangutan | unknown | Inanimate | Y |  |  |
| Play | Orangutan | unknown | Inanimate |  |  |  |
| Play | Gorilla | M to M | Social | Y |  |  |
| Play | Gorilla | unknown, infants | Social | Y |  |  |
| Approach | Chimpanzee | M to M | Social | Y |  |  |
| Play | Chimpanzee | 2 juveniles | Social | Y |  |  |
| Allogroom | Chimpanzee | M to M | Social | Y |  |  |
| Play | Chimpanzee | 2 juveniles | Social |  |  |  |
| Play | Chimpanzee | 2 juveniles | Social |  |  |  |
| Allogroom | Chimpanzee | F to F | Social | Y |  |  |
| Eat leaf | Chimpanzee | F? | Inanimate food | Y |  |  |
| Nut cracking | Chimpanzee | M | Inanimate food |  |  | Y |
| Roll stone | Chimpanzee | infant | Inanimate | Y |  |  |
| Sniff fruit | Chimpanzee | M | Inanimate food | Y |  |  |
