# Supplementary material for: Humans and great apes visually track event roles in similar ways
Source: PLoS Biol. 2024 Nov 26;22(11):e3002857. doi: 10.1371/journal.pbio.3002857 (PMC11593759; doi:10.1371/journal.pbio.3002857)
Supplement: S3 Table — (DOCX) [file pbio.3002857.s013.docx]

S3 Table. Variables coded from video stimuli

| **Variable** | **Coding description** | **Cohen’s weighted kappa** |
| --- | --- | --- |
| Time action starts^1^ | Time point at which agent starts to move. | 0.82 |
| Agent-patient movement difference | (A): agent moves more  (A/P): patient and agent have similar amount of movement  (P): patient moves more. This encompasses not just length but also intensity of movement. It should only refer to movement relevant to the action rather than anything that appears to happen prior to or independent of the action. | 0.72 |
| Direct gaze at camera | (A): agent looks at camera at any point during video  (P): patient looks at camera at any point during video  (B): both agent and patient look at camera at any point during video  (N): neither agent nor patient look at camera at any point during video | 0.82 |
| Orientation towards camera | (A): agent is positioned perpendicular to the camera at any point during video  (P): patient is positioned perpendicular to the camera at any point during video  (B): both agent and patient are positioned perpendicular to the camera at any point during video  (N): neither agent nor patient are positioned perpendicular to the camera at any point during video | 0.72 |

^1^When patient is inanimate, code NA for action start when being carried in the hand of the agent.
