# Supplementary material for: Humans and great apes visually track event roles in similar ways
Source: PLoS Biol. 2024 Nov 26;22(11):e3002857. doi: 10.1371/journal.pbio.3002857 (PMC11593759; doi:10.1371/journal.pbio.3002857)
Supplement: Attachment — Submitted filename: ComparativeEventCognition_ReviewerResponse_Aug2024.docx [file pbio.3002857.s014.docx]

**Response to reviewer comments**

Reviewer #1: I think the revised manuscript is more clearly written. I also appreciated their justification and further explanation of their statistical analyses. I still felt like the paper would benefit from some more straightforward analyses to give the reader a better intuition and more confidence in the results.

**Response: We thank the reviewer for their encouraging response. We appreciate that there are some remaining uncertainties and we have done our best to clarify these in our responses below. Regarding the analyses, please see our response to the comment below.**

I understand now that the Dirichlet model provides a measure of time looking at each AOI. I still think the percent of time looking at each AOI (calculated simply as the time at AOI/total time) without the full model would be informative. I don't insist the authors add this to the paper if the results look similar to the existing analyses, but I would be curious to see the results and this

seems very simple to calculate.

**Response: We have included the requested figure below, and also included it as Figure 8 in the supplementary material.**

I also still think that comparing heatmaps across species is important. I think the authors misunderstood my initial request - I would like to see a single heatmap per video (not time point) to quantify overall looking similarity. This would lose temporal resolution, but I believe would be a nice complement to the current time series analyses.

**Response: We appreciate the clarification of the reviewer. However, we believe that including image-based heatmaps would not add any clarity to the data given how much motion a number of our videos depict. If we were to sum or average all of these gaze points over time into a single heatmap per video, where exactly the viewer is looking would not be clear because the areas that the gaze falls on constantly changes, in most videos covering over time nearly all of the screen. As such, we don’t believe that including heatmaps would improve assessment of overall looking similarity. As an alternative solution, we have provided a figure, below and in the supplementary material, which shows the average proportion of fixations by stimulus for each species. In addition, we suggest that our current graphs actually do quantify overall looking similarity, by tracking which AOIs participants are more likely to look at over time.**

I thought the videos on OSF were somewhat confusing. In many cases, there seemed to be some panels that were not clearly labeled. E.g., video inanimate_human_compilation the bottom, left panel seems to be the infant looking behavior, but it is unclear what the other panels are.

**Response: We believe that the reviewer is referring to the supplementary_material_example_viewing, which shows compilations of different participants viewing the same videos. Descriptions specifying which panel shows what are included in the description of the file metadata next to the video (see screenshot below). For clarity, we add commas to the description to separate the description for each panel.**

Further, it seems from most of these example videos that babies are indeed looking at agents in the videos, perhaps it was just these examples, but this underscores my desire to see the above heatmap or AOI analyses.

**Response: Reviewer 2 had similar concerns about the infant data, and we have provided a more detailed response to this query there (last comment). We do however want to make it clear that we do not interpret the data as meaning that the infants never look at agents or patients, simply that they don’t track them to the same extent as adults.**

I appreciated the authors’ efforts to make the data publicly accessible, but the need to install third party software was a barrier to me exploring the data.

**Response: We thank the reviewer for highlighting this obstacle. The OSF material includes a Read.Me doc in the zip file, which explains how to set up the shiny app dashboard with three simple steps using R and Rstudio. In the spirit of open data and transparency, this allows the readers to play with the data and view the effects for different AOIs across species and variables. As a simpler option, we now also provide a url where readers can easily access the same data: https://dataplatform.evolvinglanguage.ch/eventcog_eyetracking**

**Relatedly, since reviewer 2 requested to see figures for the infant data viewing human-only footage, we have included some of the figures from the shiny app in the supplementary results (please see detailed response to R2, last comment).**

Pg 3, line 105, reference 25 (Farris 2022) seems to be incorrectly summarized, as the main finding is neural sensitivity to observed social interactions.

**Response: We appreciate that our summary of these data was not clear. We have now added the following clarification:**

***‘neural distinction of third-party social interactions does not begin to emerge until after nine months’,*** **consistent with Farris et al.’s finding for an interaction between age and condition.**

Line 754 there is a typo : Trail > Trial

**Response: Thanks for spotting this, we have corrected it.**

Reviewer #2: I appreciate the efforts that the authors have made in the revision process. Overall, the manuscript is now much clearer, and much more appropriate for publication, though I still have a few more minor recommendations:

**Response: We thank the reviewer for their positive response and additional feedback.**

There are a few places where the authors could still temper the language slightly, given that the referenced touchscreen data that bolster the agent-patient decomposition claim are not part of this manuscript:

-In line 40 (abstract), personally I feel that it would be more appropriate to say "These findings raise the possibility that event role tracking, a cognitive foundation of syntax, evolved long before language but requires time and experience to become ontogenetically available."

-Line 319 change 'indicating' to 'suggesting'

**Response: We have incorporated these changes into the manuscript.**

Given the graph produced in response to reviewer 2's concerns, I think it would be appropriate for the authors to mention that agent-patient size differences could be a strong driver of the agent bias found only in the food scenes.

**Response: We would like to reassure the reviewer that, since this is already corrected for in the models, the strong agent bias found in the food-scenes is not driven by size differences between agents and patients. We have clarified this in the manuscript on line 290:**

***‘Notably, since size of agents and patients was accounted for in the models, size cannot explain any gaze bias that we found towards agents.’***

I also agree with reviewer 1 that additional analyses on the infant data, restricted to trials in which infants were watching human videos only, would be informative. It would help clarify whether infants show a general lack of 'agent-patient viewing patterns' to complex/dynamic scenes or whether these patterns are manifest when the stimuli are more familiar. That said, the presence or lack thereof of this additional analysis does not disrupt the authors' primary findings or claims about similarities between humans and nonhuman apes.

**Response: We have included the requested figures as supplementary material (supplementary figure 7). These figures are taken from the shiny app dashboard output, and show the gaze trajectories over time for infant participants viewing both human and ape footage. As is evident in the figures, there is no apparent difference in gaze between footage of humans or apes, suggesting that their responses generalize across footage containing more familiar scenes.**
